# Willingness to Undergo Human Papillomavirus Testing Among Men Who Have Sex With Men in China Based on the Information-Motivation-Behavioral Skills Model: Online Cross-Sectional Study

**DOI:** 10.2196/85543

**Published:** 2026-06-08

**Authors:** Xin Liu, Xuelin Yang, Mohan Lyu, Zhenwei Dai, Shu Jing, You Xin, Fei Yu, Shenglan Tang, Xiaoyou Su

**Affiliations:** 1 School of Population Medicine and Public Health, Chinese Academy of Medical Sciences & Peking Union Medical College, No. 9, Dongdan 3rd Street, Dongcheng District, Beijing, Beijing, 100730, China, 86 10-65105830; 2 MRC Centre for Global Infectious Disease Analysis, Jameel Institute, School of Public Health, Imperial College London, London, United Kingdom; 3 Duke Global Health Institute, Duke University, Durham, NC, United States; 4 Peking University Sixth Hospital, Peking University Institute of Mental Health, NHC Key Laboratory of Mental Health (Peking University), National Clinical Research Center for Mental Disorders (Peking University Sixth Hospital), Beijing, China; 5 Danlan Public Welfare, Beijing, China; 6 State Key Laboratory of Respiratory Health and Multimorbidity, Beijing, China

**Keywords:** men who have sex with men, human papillomavirus, testing willingness, information-motivation-behavioral skills, associated factors

## Abstract

**Background:**

Men who have sex with men (MSM) are at high risk of human papillomavirus (HPV) infection, and HPV testing can facilitate early detection and timely intervention. However, evidence on the willingness to undergo different HPV testing modalities among MSM remains limited. The information-motivation-behavioral skills model provides a theoretical framework for understanding factors associated with the willingness to undergo HPV testing.

**Objective:**

This study aimed to investigate the current status of HPV testing and screening among MSM in China; assess their willingness to undergo professional-collected HPV testing, self-collected HPV testing, and regular HPV testing; and identify associated factors.

**Methods:**

A cross-sectional study was conducted through the Blued application (Danlan Public Welfare) from July 15, 2024, to August 2, 2024. The questionnaire collected information on participants’ sociodemographic characteristics, sexual behaviors, willingness to undergo HPV testing, HPV testing and infection history, HPV knowledge level, motivation, and behavioral skills related to HPV testing. Chi-square tests and 2-tailed *t* tests were used to compare the distribution of variables between groups. Multivariable logistic regression analyses were used to identify factors associated with willingness to undergo each HPV testing modality among MSM.

**Results:**

Among the 1080 participants recruited, 88.3% (n=954) were willing to receive professional-collected HPV testing, 83.6% (n=903) were willing to receive self-collected HPV testing, and 78.7% (n=850) were willing to engage in regular HPV testing. In multivariable analyses, willingness to undergo professional-collected HPV testing was positively associated with higher HPV knowledge (odds ratio [OR] 1.04, 95% CI 1.00‐1.08), greater perceived susceptibility (OR 1.24, 95% CI 1.01‐1.53), and higher self-efficacy (OR 1.69, 95% CI 1.14‐2.50), while it was negatively associated with perceived barriers (OR 0.61, 95% CI 0.47‐0.78). Participants who were retired or unemployed (OR 0.48, 95% CI 0.27‐0.83) and those with prior testing experience (OR 0.49, 95% CI 0.33‐0.73) were less likely to undergo self-collected HPV testing. Moreover, willingness to undergo regular HPV testing was positively associated with perceived susceptibility (OR 1.20, 95% CI 1.02‐1.40), whereas living with a partner (OR 0.54, 95% CI 0.33‐0.88) and living with family members (OR 0.69, 95% CI 0.48‐0.99) were negatively associated.

**Conclusions:**

This study suggests that willingness to undergo HPV testing among MSM in China is high, whereas prior HPV testing uptake remains low, indicating a gap between testing intention and behavior. By applying the information-motivation-behavioral skills framework to different HPV testing modalities, this study extends the previous evidence from testing willingness to understanding of testing initiation and sustained HPV screening behavior. These findings suggest that personalized health education initiatives and differentiated, privacy-sensitive, and accessible HPV testing strategies may help improve HPV testing uptake and support sustained HPV testing among MSM in China.

## Introduction

Human papillomavirus (HPV) is one of the most common sexually transmitted infections (STIs), with over 200 different types [1,2]. According to a global meta-analysis, the prevalence of HPV infection among males was estimated to be 31%, peaking in the 25‐29 years age group and remaining at a high level before the age of 50 [3]. High-risk HPV types, which are responsible for malignancies such as cervical cancer, penile cancer, and anal cancer, accounted for 21% of cases. HPV-16 was identified as the most prevalent genotype (5%), followed by HPV-6 (4%) [3]. Although HPV-6 is classified as a low-risk HPV type, it can still cause benign lesions such as genital warts. Men who have sex with men (MSM) bear a disproportionately high burden of HPV infection due to their sexual networks and behavioral characteristics. Previous research showed that the prevalence of anal high-risk HPV was 41.2% among MSM, compared to only 6.9% in other male populations [4]. Another meta-analysis of 107 international studies, including 36,773 MSM, reported pooled prevalences for anal, oral, and urethral HPV infections at 78.4%, 36.2%, and 17.3%, respectively [5].

Screening and vaccination are central strategies for preventing HPV infection and related diseases among MSM. Previous research has shown that HPV vaccines provide long-term protection, and vaccination among MSM has been reported to be cost-effective in multiple settings [6,7]. Accordingly, several countries have implemented targeted HPV vaccination programs for MSM [8,9]. In January 2025, the quadrivalent HPV vaccine (Gardasil) developed by Merck was approved in Chinese mainland for males aged 9 to 26 years, making it the first HPV vaccine indication for males in Chinese mainland [10]. This provided new opportunities for HPV prevention and control among MSM in China. However, in the short term, achieving high vaccine coverage among MSM may remain challenging due to limited vaccine supply, vaccine hesitancy, and cost-related factors [11]. Additionally, due to the sexual behavioral patterns of MSM, the herd immunity established by female HPV vaccination may provide limited protection for MSM. Moreover, some MSM also engage in sexual activity with women, which may contribute to HPV transmission to their female partners and thus have implications for cervical cancer elimination efforts in China.

HPV testing can help MSM understand their infection status, enabling them to take timely actions to reduce HPV transmission, facilitate early detection of persistent infections or lesions, and thereby reduce the risk of HPV-related diseases [12-14]. Several approaches are available for detecting HPV infections and associated lesions among MSM, including visual inspection, HPV testing, anal cytology, digital anorectal examination, and high-resolution anoscopy [15,16]. In some developed countries, regular anal screening is recommended for HIV-positive MSM to detect precancerous lesions at an early stage [17,18]. Therefore, improving HPV awareness and promoting proactive HPV testing remain critical for controlling HPV transmission among MSM. Even if HPV vaccination coverage expands in the future, regular testing and screening among MSM will still be necessary to facilitate early detection and treatment of HPV-related lesions and to reduce the disease burden.

However, implementing HPV testing and screening among MSM still faces multiple challenges. A systematic review reported that the uptake of anal cancer screening among MSM was relatively low, and limited knowledge of HPV-related risks was identified as a significant barrier, whereas greater perceived understanding was the primary facilitator [19]. Additionally, stigma and embarrassment, fear of abnormal results, and concerns about disclosure of sexual orientation in health care settings have been identified as barriers to undergoing HPV screening [20]. In China, although some institutions and community-based organizations have initiated HPV testing and screening programs for MSM, supportive programs and research evidence on HPV testing and anal screening for MSM remain limited [21]. Meanwhile, advances in HPV testing technologies have facilitated the development of self-collected HPV testing, which is considered a potential strategy for improving testing coverage among MSM due to its greater privacy, convenience, and high acceptability [22]. Therefore, it is necessary to investigate the willingness to undergo HPV testing and regular testing among MSM, to better understand their behavioral patterns and identify modifiable determinants, providing evidence for developing more targeted health education and service strategies.

This study uses the information-motivation-behavioral skills (IMB) model as its theoretical framework [23]. The IMB model categorizes behavior-influencing factors into 3 components: information (knowledge and awareness related to diseases and health behaviors), motivation (the willingness to adopt health behaviors based on personal factors and social support), and behavioral skills (the ability and skills necessary to change health behaviors). According to the IMB model, individuals with sufficient health information and motivation can initiate and maintain health behaviors through behavioral skills. The IMB model has been widely applied in research and interventions related to STIs [24-26]. A previous study used a modified IMB model to identify predictors of condom use among MSM in China, providing evidence for interventions to promote condom use [27]. Some researchers have combined mobile reminder tools with the IMB model, such as medication reminders and risk feedback, to promote pre-exposure prophylaxis adherence among MSM [28]. Another study applied an online intervention based on the IMB model to improve awareness of and willingness to receive HPV vaccination [29]. Guided by the IMB model, this study aimed to investigate the current status of HPV testing and screening among MSM in China, assess willingness to undergo professional-collected HPV testing, self-collected HPV testing, and regular HPV testing, and identify associated factors.

## Methods

### Study Design and Participants

This study was an internet-based cross-sectional survey conducted from July 15, 2024, to August 2, 2024, using the Blued platform (Supplier: Danlan public welfare), one of the most popular social networking apps for MSM in China. The technical team randomly selected 300,000 users nationwide who had logged into Blued within the past month and sent them a recruitment poster. Interested users could click the link or scan the QR code on the poster to access the questionnaire. All participants were required to provide informed consent before completing the questionnaire. The inclusion criteria were as follows: (1) aged 18 years and older; (2) male at birth; (3) had anal intercourse with a male partner in the past 6 months; and (4) consented to participate in this study. Each IP address was allowed to submit the questionnaire only once, and all items were required to be completed before submission.

### Study Size

The sample size was estimated using the formula for cross-section studies (Eq. 1). Based on our pilot survey, the willingness to undergo HPV testing among MSM was 95% (57/60). With a 95% CI and an allowable absolute error set at 1.5%, the required sample size was 811. Allowing for an anticipated 20% invalid response rate, the minimum required sample size was 1014.


(1) $$n=\frac{Z_{1-\alpha /2}^{2}p(1-p)}{d^{2}}$$

Here *n* is the sample size, *Z* is the *z*-score (the critical value from the standard normal distribution), *p* is the anticipated or estimated population proportion, and *d* is precision or margin of error.

### Measurements

The survey questionnaire used in this study was designed by a panel consisting of epidemiologists, physicians, statisticians, nongovernmental organization workers, and linguists, based on the findings of prior research and preliminary studies [24,30]. The questionnaire development followed a 4-step process. First, a targeted review of previous studies on HPV testing, HPV vaccination, and the IMB framework was conducted to identify candidate items. These items were adapted and translated to form an initial item pool. Second, we recruited 28 MSM and conducted semistructured interviews guided by the IMB model. Themes derived from the qualitative analysis were used to refine the constructs and revise item wording. Third, the draft questionnaire was reviewed by the panel to assess content relevance, clarity, and scientific accuracy. Fourth, a pilot survey was conducted among MSM to evaluate feasibility, comprehensibility, and skip logic, and the questionnaire was finalized accordingly.

The questionnaire included sections on sociodemographic information, sexual behavioral characteristics, health status, the constructs of the IMB model, including scales and items assessing the HPV-related knowledge, motivation, and behavioral skills regarding HPV testing, and MSM’s willingness to undergo HPV testing. In total, 1094 questionnaires were completed. After quality control, 14 questionnaires were excluded, leaving 1080 valid questionnaires for analysis. Exclusions were made for the following reasons: response time of less than 200 seconds (n=10), residing outside of China in the past 6 months (n=3), and inconsistent or nonsensical responses (n=1).

### Sociodemographic Characteristics

We collected sociodemographic information on participants, including age, gender at birth, gender identity, sexual orientation, city of residence, educational level, current employment status, monthly personal income, and cohabitation status. Sexual behavioral characteristics were also collected, including sexual role, number of sexual partners, whether they had engaged in anal sex, oral sex, commercial sex, or group sex in the past 6 months, and whether they had sex with regular partners, temporary partners, or female partners in the past 6 months. Health-related variables included circumcision status, history of HIV testing, HIV status, history of STIs, and history of HPV testing.

### Willingness to Undergo HPV Testing

Participants were first introduced to professional-collected HPV testing. “*Professional-collected HPV testing requires visiting a testing site (such as a hospital), where a professional uses a swab to collect samples from the anus, coronal sulcus (glans penis), and/or oral cavity.*” Participants were then asked whether they would be willing to undergo professional-collected HPV testing if the test were free. Subsequently, participants were introduced to self-collected HPV testing. “*Self-collected HPV testing requires users to obtain a testing kit online, follow the instructions to collect samples from the anus, coronal sulcus (glans penis), and/or oral cavity by themselves, and then send the samples in a preservation tube to a designated lab for testing.*” Participants were then asked whether they would be willing to undergo self-collected HPV testing if it were free. Among MSM who were willing to undergo at least 1 type of HPV testing, further inquiries were made regarding their reasons for acceptance. Moreover, all participants were asked about their preferred testing frequency if testing were needed (response options: 1=every 6 months, 2=annually, 3=only when necessary, and 4=other). Participants who chose every 6 months, annually, or any other specific frequency were classified as having an intention for regular HPV testing. MSM who were unwilling to undergo either type of HPV testing were asked about their reasons for refusal.

### Information of HPV Testing

HPV knowledge among MSM was assessed using a scale developed by Waller [31] in 2013. This scale includes general HPV knowledge subscale (GK), HPV testing knowledge subscale (TK), and HPV vaccine knowledge subscale (VK), which assess individuals’ knowledge of HPV. It has been applied in Chinese populations and has demonstrated good reliability among college students [32]. In this study, we used the GK including 16 items to measure participants’ general understanding of HPV and TK consisting of 6 items to assess knowledge related to HPV testing. Each item offers 3 response options: “True,” “False,” and “Don’t know”. Correct answers were scored as 1, whereas incorrect or “Don’t know” responses were scored as 0. Higher scores indicated greater knowledge level of HPV. In this study, the Cronbach *α* for GK and TK were 0.927 and 0.839, respectively.

### Motivation for HPV Testing

Motivation for HPV testing was assessed using 17 items across 5 domains, adapted from previous studies and our unpublished qualitative study (Table S1 in Multimedia Appendix 1) [24,33]. Perceived susceptibility was assessed by 3 items evaluating the perceived risk of HPV infection and related diseases among participants. Perceived severity was measured using 3 items that assessed participants’ perceived seriousness of HPV-related diseases. Perceived benefits were evaluated through 3 items that gauged participants’ perceived health benefits of HPV testing. Perceived barriers included 5 items assessing perceived difficulties that might be encountered during decision-making or action-taking processes. Subjective norms were assessed by 3 items measuring perceived support from family and peers for the participants’ decision to undergo HPV testing. All items were rated on a 5-point Likert scale (1=Strongly disagree, 2=Disagree, 3=Neutral, 4=Agree, and 5=Strongly agree). Higher scores indicated stronger motivation for HPV testing, except for perceived barriers, for which higher scores indicated greater perceived barriers. In this study, the Cronbach *α* of motivation for HPV testing was 0.850.

### Behavioral Skills for HPV Testing

Behavioral skills for HPV testing were assessed through 5 items across 3 domains, based on previous studies and our unpublished qualitative study (Table S1 in Multimedia Appendix 1) [24,34]. Decision-making was assessed by 1 item evaluating participants’ agency in deciding whether to undergo HPV testing. Self-efficacy contained 2 items assessing participants’ confidence in overcoming potential difficulties to take action. Objective skills consisted of 2 items evaluating participants’ skills and abilities necessary for undergoing HPV testing. All items were rated on a 5-point Likert scale (1=Strongly disagree, 2=Disagree, 3=Neutral, 4=Agree, and 5=Strongly agree). Higher scores indicated greater behavioral skills for HPV testing. In this study, the Cronbach *α* of behavioral skills for HPV testing was 0.884.

### Bias

Several measures were taken to address potential sources of bias. The survey was administered anonymously, which may have reduced social desirability bias when participants reported sensitive sexual behaviors and STI history. For questions on sexual behaviors, a 6-month recall period was used to improve recall accuracy. Explanations of professional-collected and self-collected HPV testing were provided to reduce differences in participants’ understanding of the testing procedures. All questionnaire items were mandatory to ensure completeness of responses and avoid missing data. No financial compensation was provided to participants, which may have reduced participation driven by monetary incentives. However, because this was a cross-sectional survey based on self-reported data, bias could not be fully eliminated.

### Statistical Analysis

In this study, the distribution of variables between participants who were willing and unwilling to undergo the HPV testing was reported using means and SDs for continuous variables and frequencies and percentages (%) for categorical variables. Chi-squared tests and 2-tailed *t* tests were used to compare differences in variable distributions between groups. To reduce the risk of excluding potentially important predictors, variables with a *P* value of <.10 in the univariate analysis, as well as variables with professional relevance, were included in the multivariable logistic regression analysis. Multivariable logistic regressions using the Enter method, in which all selected covariates were entered into the model simultaneously, were used to identify factors associated with willingness to undergo HPV testing among MSM. Adjusted odds ratios [OR] and their corresponding 95% CI were calculated. Multicollinearity was assessed using the variance inflation factor, and all variance inflation factor values were <5, indicating no significant multicollinearity in the model. Statistical significance was assessed using 2-sided tests with a *P* value <.05 for all analyses.

### Ethical Considerations

This study received ethics approval from the institutional ethics committee of the Chinese Academy of Medical Sciences and Peking Union Medical College (CAMS&PUMC-IEC-2022-078). The study followed the Strengthening the Reporting of Observational Studies in Epidemiology (STROBE) guidelines [35]. Digital informed consent was obtained from all participants. All data used in this study were deidentified, and no participants were identifiable in any images included in the manuscript or supplementary material. Participants received no compensation.

## Results

### Sociodemographic Characteristics of Participants

This study recruited 1080 participants, with a mean age of 31.14 (SD 9.45) years. Most of them were identified as male participants (n=1002, 92.8%), homosexual (n=825, 76.4%), were employed full-time (n=840, 77.8%), and lived in the northern (n=258, 23.9%) and eastern (n=258, 23.9%) regions of China. Among the participants, 34.1% (n=368) of MSM reported monthly incomes ranging from CN ¥3000 to ¥6999 (US $435-$1015), and 42.6% (n=460) lived alone (Table 1).

**Table 1.** Baseline characteristics of men who have sex with men (MSM) recruited online in China, 2024.

Variables	Overall (N=1080)
Age (y), mean (SD)	31.14 (9.45)
Gender identity, n (%)	
Male	1002 (92.8)
Transgender woman	78 (7.2)
Region, n (%)	
Northeast	73 (6.7)
North	258 (23.9)
Central	135 (12.5)
East	258 (23.9)
South	177 (16.4)
Southwest	125 (11.6)
Northwest	54 (5)
Education level, n (%)	
High school and below	210 (19.4)
High school above	870 (80.6)
Current employment status, n (%)	
Full-time	840 (77.8)
Student	163 (15.1)
Unemployed or retired	77 (7.1)
Personal monthly income (CN ¥; CN ¥1=US $0.14)	
<3000	232 (21.5)
3000‐6999	368 (34.1)
7000‐9999	191 (17.7)
≥10,000	289 (26.7)
Cohabitation status, n (%)	
Alone	460 (42.6)
Partner	118 (10.9)
Family	318 (29.5)
Friends or roommates	172 (15.9)
Others	12 (1.1)
Sexual orientation, n (%)	
Homosexuality	825 (76.4)
Bisexuality	190 (17.6)
Heterosexual and others	65 (6)
Sex role, n (%)	
Receptive	366 (33.9)
Versatile	393 (36.4)
Insertive	321 (29.7)
Number of sexual partners, n (%)	
1	354 (32.8)
≥2	726 (67.2)
Condom usage frequency during anal sex, n (%)	
Every time	531 (49.2)
Often use	210 (19.4)
Occasionally use	252 (23.3)
Never use	87 (8.1)
Performed oral sex in the past 6 mo, n (%)	
No	250 (23.1)
Yes	830 (76.9)
Received oral sex in the past 6 mo, n (%)	
No	423 (39.2)
Yes	657 (60.8)
Condom use frequency during oral sex, n (%)	
Every time	33 (3.1)
Often use	12 (1.1)
Occasionally use	65 (6)
Never use	926 (85.7)
No oral sex before	44 (4.1)
Had sex with regular partners in the past 6 mo, n (%)	
No	464 (43)
Yes	616 (57)
Had sex with temporary partners in the past 6 mo, n (%)	
No	465 (43.1)
Yes	615 (56.9)
Had commercial sex in the past 6 mo, n (%)	
No	1013 (93.8)
Yes	67 (6.2)
Had group sex in the past 6 mo, n (%)	
No	897 (83.1)
Yes	183 (16.9)
Had sex with female in the past 6 mo, n (%)	
No	962 (89.1)
Yes	118 (10.9)
Had a circumcision, n (%)	
No	861 (79.7)
Yes	219 (20.3)
Self-reported HIV status, n (%)	
Positive	105 (9.7)
Negative	790 (73.2)
Unknown	185 (17.1)
History of STIs^a^, n (%)	
No	702 (65)
Yes	378 (35)
Information (score), mean (SD)	
Knowledge of HPV^b^	13.45 (5.40)
Motivation (score), mean (SD)	
Perceived susceptibility	3.23 (1.02)
Perceived severity	3.79 (0.99)
Perceived benefits	4.24 (0.82)
Perceived barriers	3.33 (0.94)
Subjective norms	3.44 (0.78)
Behavioral skills (score), mean (SD)	
Decision-making	4.11 (0.88)
Self-efficacy	3.98 (0.82)
Objective skills	3.83 (0.89)

a STI: sexually transmitted infection.

b HPV: human papillomavirus.

### Characteristics of Sexual Behaviors and Health Status

In the past 6 months, all 1080 participants had engaged in anal sex with men: 36.4% (n=393) of them were versatile, 49.2% (n=531) consistently used condoms, and 95.9% (n=1036) had engaged in oral sex. Additionally, 76.9% (n=830) reported that they had performed oral sex and 60.8% (n=657) had received oral sex in the past 6 months. However, only 3.1% (n=33) reported using condoms during oral sex. Furthermore, 57% (n=616) of the MSM had sex with regular partners, 56.9% (n=615) with temporary partners, 6.2% (67/1080) had commercial sex, 10.9% (n=118) had sex with females, 16.9% (n=183) had group sex, and 67.2% (n=726) had 2 or more sexual partners. Additionally, 20.3% (n=219) had undergone circumcision, 9.7% (n=105) reported being HIV-positive, and 35% (n=378) had a history of STIs (Table 1).

### Dimensions of the IMB Model

In this study, participants had a mean HPV knowledge score of 13.45 (SD 5.40). Regarding motivation for HPV testing, the mean scores were 3.23 (SD 1.02) for perceived susceptibility, 3.79 (SD 0.99) for perceived severity, 4.24 (SD 0.82) for perceived benefits, 3.33 (SD 0.94) for perceived barriers, and 3.44 (SD 0.78) for subjective norms. In terms of behavioral skills for HPV testing, the mean scores were 4.11 (SD 0.88) for decision-making, 3.98 (SD 0.82) for self-efficacy, and 3.83 (SD 0.89) for objective skills (Table 1).

### Characteristics and Reasons for HPV Screening Among MSM

Among participants, 35.2% (380/1080) had previously undergone HPV screening and 23.3% (252/1080) had undergone HPV testing, with 43.3% of them (109/252) testing positive. Additionally, 17.8% (192/1080) had undergone an acetic acid test, 16.9% (182/1080) had undergone anoscopy, 5.5% (59/1080) had undergone anal cytology, and 22.6% (244/1080) reported a history of genital warts. This study investigated the reasons for HPV screening among participants with an HPV screening history. The most common reasons were “*appearance or suspected appearance of HPV-related symptoms*” (224/380, 58.9%), “*voluntary proactive testing*” (211/380, 55.5%), “*engagement in high-risk sexual behavior*” (96/380, 25.3%), “*recommendation by healthcare professionals*” (57/380, 15%), “*had sex with an HPV-positive individual*” (33/380, 8.7%), and “*participation in an HPV vaccine clinical trial*” (13/380, 3.4%) (Table 2 and Table S2 in Multimedia Appendix 1).

**Table 2.** HPV^a^ testing history and HPV infection status among men who have sex with men (MSM) recruited online in China, 2024.

Variables	Total (N=1080)	HIV status	
		Negative or unknown (n=975)	Positive (n=105)
Ever received any HPV-related examination, n (%)			
No	700 (64.8)	639 (65.5)	61 (58.1)
Yes	380 (35.2)	336 (34.5)	44 (41.9)
Ever received HPV DNA testing, n (%)			
No	828 (76.7)	751 (77)	77 (73.3)
Yes	252 (23.3)	224 (23)	28 (26.7)
Ever received acetic acid test, n (%)			
No	888 (82.2)	806 (82.7)	82 (78.1)
Yes	192 (17.8)	169 (17.3)	23 (21.9)
Ever received anoscopy, n (%)			
No	898 (83.1)	816 (83.7)	82 (78.1)
Yes	182 (16.9)	159 (16.3)	23 (21.9)
Ever received anal cytology, n (%)			
No	1021 (94.5)	921 (94.5)	100 (95.2)
Yes	59 (5.5)	54 (5.5)	5 (4.8)
HPV test result (n=252), n (%)			
Positive	109 (43.3)	93 (41.5)	16 (57.1)
Negative	143 (56.7)	131 (58.5)	12 (42.9)
History of genital warts, n (%)			
No	836 (77.4)	779 (79.9)	57 (54.3)
Yes	244 (22.6)	196 (20.1)	48 (45.7)

a HPV: human papillomavirus.

### Willingness to Undergo HPV Testing and Regular HPV Testing

The overall willingness to undergo HPV testing in this study was 97% (1048/1080). Of the participants, 74.9% (n=809) were willing to undergo both types of HPV testing, 13.4% (n=145) were willing to undergo only the professional-collected HPV testing, and 8.7% (n=94) were willing to undergo only the self-collected testing. The willingness to undergo the professional-collected HPV testing was 88.3% (n=954), the willingness to undergo self-collected HPV testing was 83.6% (n=903), and the willingness to engage in regular HPV testing was 78.7% (n=850).

### Reasons for Unwillingness to Undergo HPV Testing

In this study, among 1080 participants, 32 (3%) expressed their unwillingness to undergo either type of HPV testing. The 3 most common reasons were as follows: “*I am reluctant to expose my genital area to others*” (22/32, 68.8%); “*I am concerned that sampling myself may affect the accuracy of the results*” (20/32, 62.5%); and “*I am concerned about the leakage of my private information*” (18/32, 56.3%). Additionally, 94 (8.7%) participants were unwilling to undergo the professional-collected HPV testing. The 3 most common reasons were as follows: “*I am reluctant to expose my genital area to others*” (65/94, 69.1%); “*I am concerned about the leakage of my private information*” (61/94, 64.9%); and “*Undergoing HPV testing may bring negative comments to me*” (45/94, 47.9%). Furthermore, 145 (13.4%) participants expressed unwillingness to undergo self-collected HPV testing, with the 3 most common reasons being as follows: “*I am concerned that sampling myself may affect the accuracy of the results*” (119/145, 82.1%), “*I suspect the accuracy of self-collected HPV testing*” (97/145, 66.9%), and “*I think the testing process is complicated*” (62/145, 42.8%) (Table S3 in Multimedia Appendix 1).

### Factors Associated With Willingness to Undergo Professional-Collected HPV Testing

**Figure 1.**
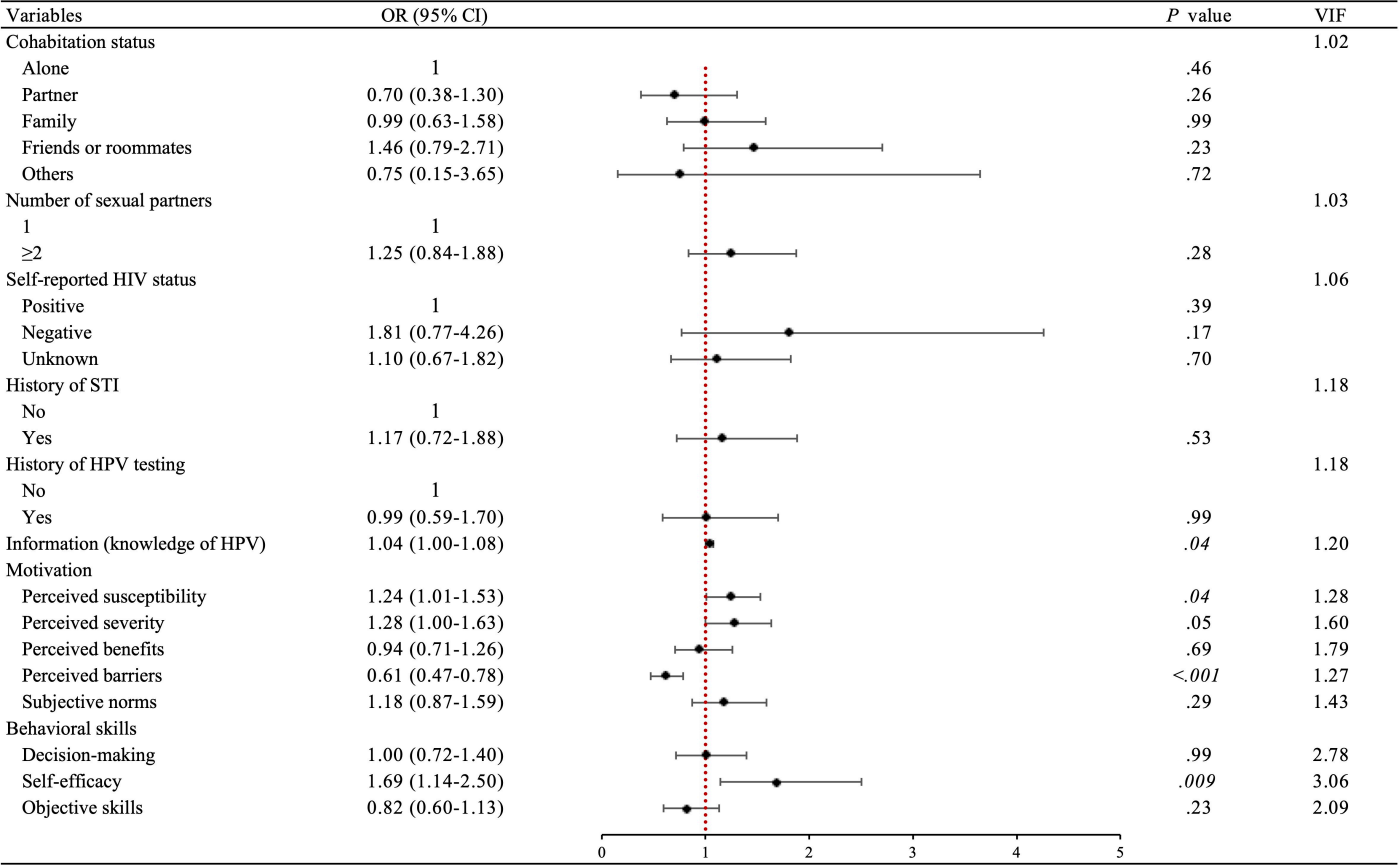
Factors influencing willingness for professional-collected human papillomavirus (HPV) testing among men who have sex with men (MSM) in China (N=1080). OR: odds ratio; STI: sexually transmitted infection; VIF: variance inflation factor.

The factors associated with willingness to undergo professional-collected HPV testing are presented in Table S4 of Multimedia Appendix 1. The multivariable logistic regression analysis showed that participants with greater HPV knowledge (OR 1.04, 95% CI 1.00‐1.08), perceived susceptibility (OR 1.24, 95% CI 1.01‐1.53), and self-efficacy (OR 1.69, 95% CI 1.14‐2.50) had a higher intention to undergo the professional-collected HPV test. Conversely, those with higher perceived barriers (OR 0.61, 95% CI 0.47‐0.78) were less likely to be willing to undergo the professional-collected HPV test (Figure 1).

### Factors Associated With Willingness to Undergo Self-Collected HPV Testing

**Figure 2.**
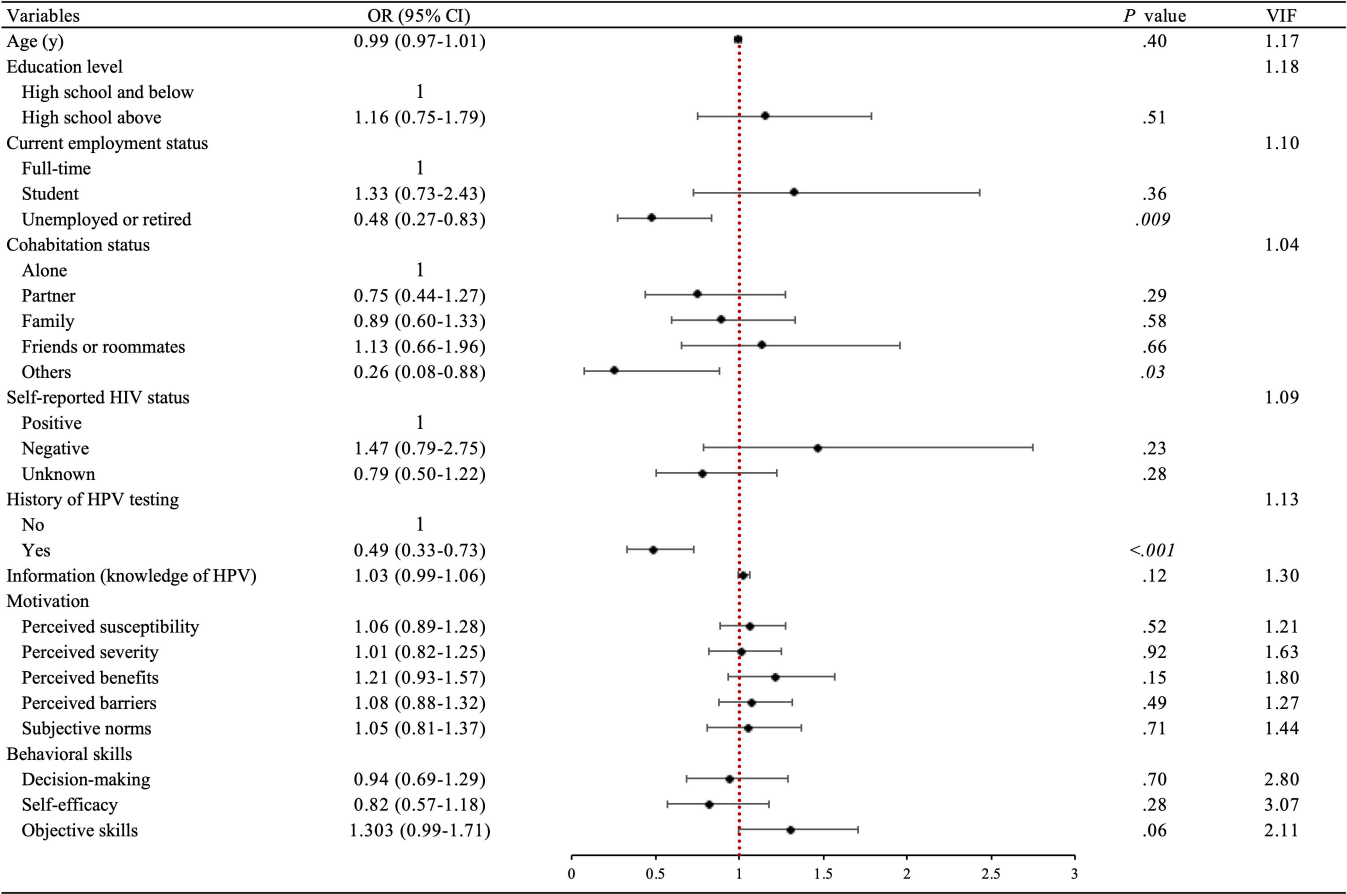
Factors influencing willingness for self-collected human papillomavirus (HPV) testing among men who have sex with men in China (N=1080). OR: odds ratio; VIF: variance inflation factor.

The factors associated with the willingness to undergo self-collected HPV testing are presented in Table S5 of Multimedia Appendix 1. The multivariable logistic regression analysis showed that retired and unemployed participants (OR 0.48, 95% CI 0.27‐0.83) were less likely to be willing to undergo the self-collected HPV test compared with those with full-time jobs. Additionally, compared with MSM who had never undergone HPV testing, those who had previously undergone testing were less willing to undergo self-collected testing (OR 0.49, 95% CI 0.33‐0.73) (Figure 2).

### Factors Associated With Willingness to Undergo Regular HPV Testing

**Figure 3.**
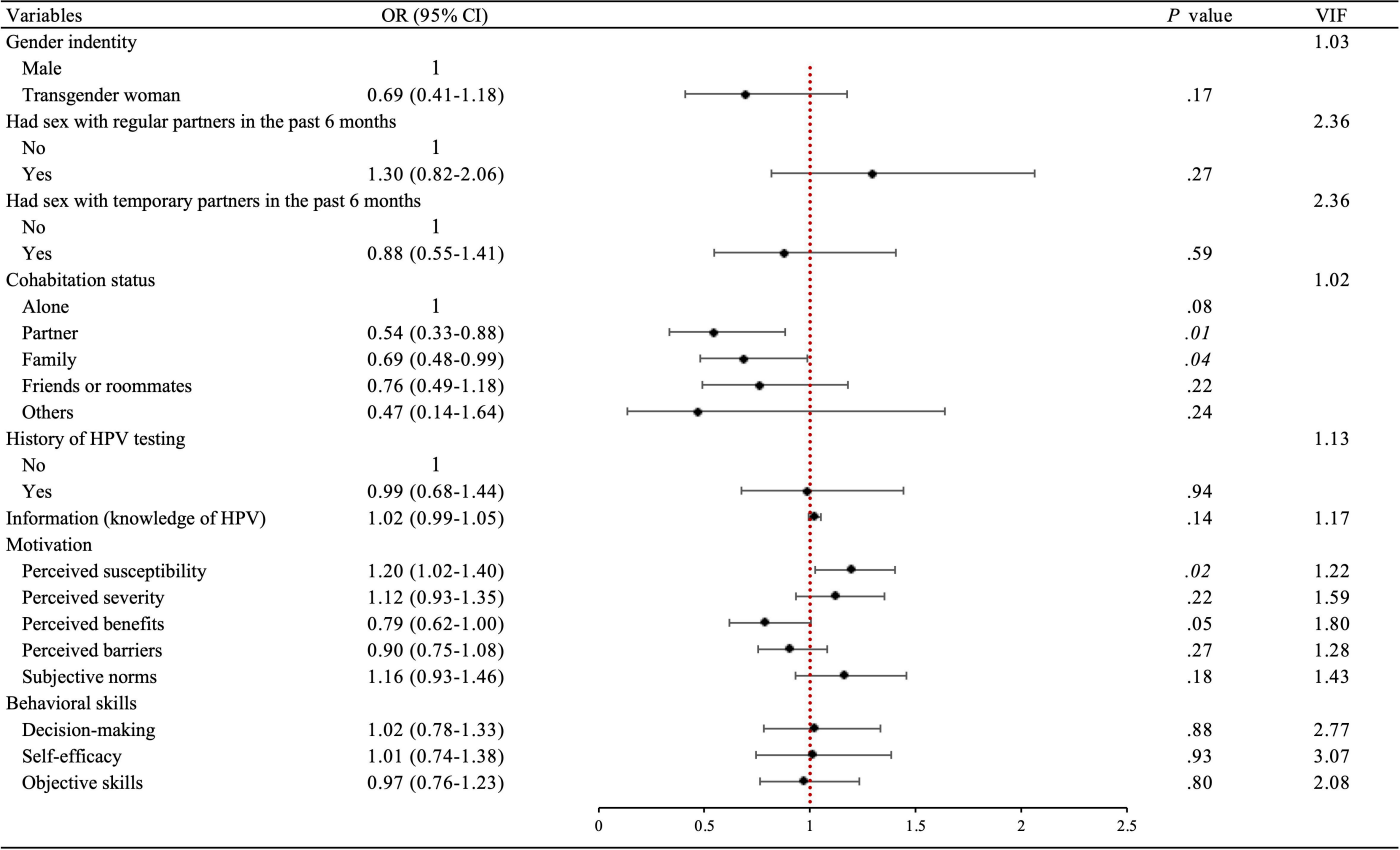
Factors influencing willingness for regular human papillomavirus (HPV) testing among men who have sex with men in China (N=1080). OR: odds ratio; VIF: variance inflation factor.

In this study, we explored factors associated with the willingness to undergo regular HPV testing (Table S6 in Multimedia Appendix 1). The results of the multivariable logistic regression analysis indicated that participants with higher perceived susceptibility (OR 1.20, 95% CI 1.02‐1.40) were more likely to accept regular HPV testing. However, MSM living with a partner (OR 0.54, 95% CI 0.33‐0.88) and those living with family members (OR 0.69, 95% CI 0.48‐0.99) were less likely to undergo regular HPV testing (Figure 3).

## Discussion

### Principal Findings

This study investigated willingness to undergo HPV testing and its associated factors among MSM in China. Although most participants were willing to undergo HPV testing, the prior HPV testing uptake was only 23.3% (252/1080), which was similar to that reported in a study conducted in Hong Kong from 2019 to 2020 (67/350, 19.1%) [30]. However, the main reason for undergoing HPV testing was the presence or suspected presence of HPV-related symptoms, indicating a significant gap in HPV prevention among MSM. The analysis guided by the IMB model indicated that the gap between high willingness and low uptake of HPV testing among MSM reflects insufficient knowledge of HPV prevention, as well as multiple barriers that hinder the translation of willingness into actual testing behavior. These findings provide evidence for the development of more targeted public health intervention strategies.

In line with other studies [36,37], this study found that the level of HPV-related knowledge among MSM in China was relatively low. According to previous research, higher levels of knowledge are associated with a stronger intention to undergo HPV screening [38]. Individuals with greater HPV knowledge are generally more health-conscious and better able to understand the benefits of HPV testing, which is associated with a higher willingness to undergo HPV testing. Furthermore, due to sociocultural factors that contribute to unequal access to health information, many MSM lack sufficient awareness of the harms of HPV infection [39]. This was reflected in an underestimation of the threat posed by HPV to male health and inadequate recognition of the role of high-risk sexual behaviors in facilitating HPV transmission [40]. An intervention study in the United States showed that, after receiving HPV-related health education, participants’ awareness that “HPV infection may cause anal cancer” increased, and their willingness to seek HPV testing improved [41]. These findings suggest that health authorities should work together with community-based organizations to deliver targeted health education for MSM and make greater use of internet-based platforms and new media technologies to expand outreach and improve HPV-related knowledge among MSM.

Perceived susceptibility was significantly associated with the willingness to undergo HPV testing and regular testing among MSM [42]. Previous research has shown that perceived risk of HIV infection significantly increases the intention to undergo regular testing among MSM [43]. Similarly, when MSM recognize that their sexual behaviors place them at higher risk of HPV infection, they are more likely to actively seek testing to identify their infection status [44]. Additionally, regular screening for HPV and anal cancer has been shown to increase life expectancy among MSM and is considered a cost-effective intervention [45]. Previous research also suggests that individuals with a better understanding of their potential health risks are more likely to engage in self-protective behaviors [46]. Therefore, MSM with higher perceived susceptibility to HPV tend to be more aware of their infection risk, show greater concern about their health status, and are more likely to initiate and maintain HPV testing behaviors [47]. This finding suggests that HPV-related services could be integrated with HIV prevention and other sexual health services within a broader STI prevention framework, which may provide a more effective approach to improving testing uptake and reducing STI transmission in this population.

In addition, perceived barriers also affect the willingness to undergo HPV testing among MSM. According to structural barriers theory, limited access to health care services and sociocultural pressures can significantly inhibit health behaviors [48]. A study on HIV testing willingness among MSM found that concerns about privacy leakage led 30% of respondents to avoid testing [49]. Moreover, STIs testing can cause stigma, which may increase their perceived barriers and reduce their willingness to undergo HPV testing [50]. When MSM are faced with issues such as shame, inadequate privacy protection, and stigma during the testing process, they may become more reluctant to undergo professional-collected HPV testing. Therefore, exploring the feasibility of delivering confidential and MSM-friendly professional-collected HPV testing, or professionally supported self-collected HPV testing, through community-based organizations may be a valuable strategy. Such service models may better meet the diverse needs of MSM and help improve testing coverage and adherence.

Improving self-efficacy among MSM is beneficial for promoting testing [51]. Previous research has shown that women with higher self-efficacy are more likely to undergo HPV testing [52]. MSM with greater awareness of self-protection and stronger confidence in HPV testing are more likely to overcome barriers associated with professional-collected HPV testing. Moreover, self-collected HPV testing provides a rapid, confidential, and cost-effective option [53]. Therefore, comprehensive efforts are needed to strengthen social advocacy that raises public awareness of HPV infection and reduces discrimination and stigma toward MSM. A meta-analysis showed that offering home-based self-collection kits contributes to higher participation rates compared with clinic-based professional sampling [54]. Self-sampling HPV testing has also been found to be acceptable and feasible among MSM [30]. Optimizing professional-collected HPV testing procedures to ensure greater privacy protection and promoting self-sampling HPV testing may help reduce psychological resistance and improve testing coverage among MSM [55].

In this study, retired or unemployed participants were less likely to be willing to undergo self-collected HPV testing compared with those who were employed. This may be related to lower occupational stability, which is often associated with reduced access to health care services [56]. Retired or unemployed individuals may face unstable income or insufficient health insurance coverage, making them more reliant on free or low-cost medical services [57]. Although self-collected HPV testing offers greater privacy, it typically requires purchasing test kits, which may impose an additional financial burden on retired and unemployed MSM [58]. Additionally, these groups are less likely to obtain testing information through online platforms or social media, leading to lower awareness and acceptance of self-collected HPV testing [59]. Therefore, governments and communities should consider providing subsidized or free HPV testing services or kits for retired and unemployed MSM. Targeted health education initiatives delivered through community for older adults may help improve awareness and acceptance of HPV testing [60].

This study extends existing evidence on HPV prevention among MSM by providing a theory-informed assessment of HPV testing willingness. First, this study distinguished between professional-collected and self-collected HPV testing, which helped identify modality-specific factors associated with different testing approaches. Second, by including regular HPV testing, this study extended the focus from one-time testing acceptance to sustained HPV screening behavior among MSM. Third, by applying the IMB model, this study interpreted testing initiation and sustained HPV testing behavior through the dimensions of information, motivation, and behavioral skills, providing a theoretical basis for designing differentiated interventions.

### Limitations

This study has several limitations. First, participants were recruited using online convenience sampling, which may have introduced selection bias and limited the representativeness of the sample, as MSM with lower digital access and health literacy may have been underrepresented. Second, the data were collected through self-administered questionnaires, which may have resulted in recall bias and social desirability bias for sensitive information such as sexual behaviors and history of STIs. Third, motivation and behavioral skills related to HPV testing were assessed using self-developed, nonvalidated items, which may not have fully captured all relevant domains. Fourth, because this was a cross-sectional study, causal relationships between outcomes and associated factors could not be established. Accordingly, the findings should be interpreted with caution, and further longitudinal studies are needed to validate and extend these results.

### Conclusion

This study investigated the willingness of MSM in China to undergo different HPV testing modalities and, based on the IMB model, explored the gap between willingness to undergo HPV testing and actual testing uptake in this population. The findings support the applicability of the IMB model in explaining willingness to undergo HPV testing among MSM and provide a basis for future research and strategy development. Our findings suggest that personalized health education should be strengthened through internet platforms and new media technologies to improve HPV knowledge among MSM. Meanwhile, diversified and privacy-sensitive testing service models should be explored to enhance the convenience and accessibility of HPV testing. These strategies may help translate testing willingness into actual testing behavior and promote the sustainable implementation of HPV testing services among MSM in China.

## Supplementary material

10.2196/85543 Multimedia Appendix 1 Items assessing motivation and behavioral skills related to human papillomavirus (HPV) testing, reasons for HPV screening and unwillingness to undergo HPV testing, and univariate analyses of factors associated with willingness to undergo professional-collected HPV testing, self-collected HPV testing, and regular HPV testing among men who have sex with men (MSM) in China.

## Abbreviations

GK: general HPV knowledge subscale
HPV: human papillomavirus
IMB: information-motivation-behavioral skills
MSM: men who have sex with men
OR: odds ratio
STI: sexually transmitted infection
STROBE: Strengthening the Reporting of Observational Studies in Epidemiology
TK: HPV testing knowledge subscale
VK: HPV vaccine knowledge subscale
