# Supplementary material for: Willingness to Undergo Human Papillomavirus Testing Among Men Who Have Sex With Men in China Based on the Information-Motivation-Behavioral Skills Model: Online Cross-Sectional Study
Source: JMIR Public Health Surveill. 2026 Jun 8;12:e85543. doi: 10.2196/85543 (PMC13245549; doi:10.2196/85543)
Supplement: Multimedia Appendix 1 [file publichealth-v12-e85543-s001.docx]

| Supplementary Table1 Items of motivation and behavioral skills measures included in the questionnaire | | | | |
| --- | --- | --- | --- | --- |
| Domain | | Item | Item origin | Source |
| Motivation | Perceived susceptibility | I think I am at risk of HPV infection. | Adopted | [21], [30] |
|  |  | I think I am at risk of developing genital warts. | Modified | [21] |
|  |  | I think I am at risk of developing anal cancer. | Modified | [21] |
|  | Perceived severity | When I think that I might be infected with HPV, I feel afraid. | Adopted | [21] |
|  |  | Developing genital warts would be devastating for me. | Modified | [21] |
|  |  | Developing anal cancer would be devastating for me. | Modified | [21] |
|  | Perceived benefits | HPV testing can help me understand my own infection status. | New | Unpublished qualitative research |
|  |  | Regular HPV testing can help me prevent HPV-related diseases. | New | Unpublished qualitative research |
|  |  | HPV testing is a way to protect people who are important to me. | New | Unpublished qualitative research |
|  | Perceived barriers | The sample collection process for HPV testing would make me feel embarrassed. | New | Unpublished qualitative research |
|  |  | I am concerned that HPV testing is expensive. | Modified | [21], [31] |
|  |  | I am concerned about leakage of my private information. | Modified | [21] |
|  |  | I doubt the accuracy of HPV testing. | New | Unpublished qualitative research |
|  |  | Having HPV testing may bring me negative comments and consequences. | Modified | [21], [30], [31] |
|  | Subjective norms | People around me would support me in getting HPV testing. | Modified | [21], [30], [31] |
|  |  | Someone around me has had HPV testing. | Modified | Unpublished qualitative research |
|  |  | I am influenced by the experiences or advice of people around me. | Modified | [21], [30], [31] |
| Behavioral Skills | Self-decision | I can decide for myself whether to get HPV testing. | New | Unpublished qualitative research |
|  | Self-efficacy | If I want, I can complete HPV testing. | Modified | Unpublished qualitative research |
|  |  | Even if I have to pay, I am willing to get HPV testing. | Modified | [21], [31] |
|  | Objective skills | It is easy for me to find information about HPV testing. | Modified | [21] |
|  |  | I believe I can clearly express my purpose and needs for testing. | New | Unpublished qualitative research |

| Supplementary Table 2 Reasons for HPV screening among MSM with HPV screening history in China (N=380) | |
| --- | --- |
| Reasons | n (%) |
| Voluntary proactive testing. | 211 (55.5) |
| Appearance or suspected appearance of HPV-related symptoms. | 224 (58.9) |
| Engagement in high-risk sexual behavior. | 96 (25.3) |
| Recommendation by healthcare professionals. | 57 (15.0) |
| Had sex with an HPV-positive individual. | 33 (8.7) |
| Participation in an HPV vaccine clinical trial. | 13 (3.4) |
| Others. | 7 (1.8) |

| Supplementary Table 3 Reasons for unwillingness to undergo HPV testing among MSM in China | |
| --- | --- |
| Reasons | n (%) |
| Unwillingness to undergo any HPV testing (n=32) |  |
| I do not have any risk of HPV infection. | 13 (40.6) |
| I am concerned about the leakage of my private information. | 18 (56.3) |
| Undergoing HPV testing may bring negative comments to me. | 7 (21.9) |
| I think the testing process is complicated. | 11 (34.4) |
| I am reluctant to expose my genital area to others. | 22 (68.8) |
| I suspect the accuracy of routine HPV Testing. | 11 (34.4) |
| I am concerned that sampling myself may affect the accuracy of the results. | 20 (62.5) |
| I suspect the accuracy of self-collected HPV testing. | 14 (43.8) |
| Others (expensive, do not want to register with real name). | 2 (6.3) |
| Unwillingness to undergo professional-collected HPV testing (n=94) |  |
| I am reluctant to expose my genital area to others. | 65 (69.1) |
| I suspect the accuracy of professional-collected HPV testing. | 16 (17.0) |
| I am concerned about the leakage of my private information. | 61 (64.9) |
| Undergoing HPV testing may bring negative comments to me. | 45 (47.9) |
| I think the testing process is complicated. | 41 (43.6) |
| Others (expensive). | 1 (1.1) |
| Unwillingness to undergo self-collected HPV testing (n=145) |  |
| I am concerned that sampling myself may affect the accuracy of the results. | 119 (82.1) |
| I suspect the accuracy of self-collected HPV testing. | 97 (66.9) |
| I am concerned about the leakage of my private information. | 53 (36.6) |
| Undergoing HPV testing may bring negative comments to me. | 23 (15.9) |
| I think the testing process is complicated. | 62 (42.8) |
| Others (expensive, dislike). | 2 (1.4) |

| Supplementary Table 4 Univariate analysis of factors influencing willingness for professional-collected HPV testing among MSM in China (N=1080) | | | | |  |
| --- | --- | --- | --- | --- | --- |
| Variables | willingness for professional-collected HPV testing | | Statistics | | |
|  | Unwillingness | willingness | *χ^2^ or t* | *P value* | *df* |
|  | (n=126) | (n=954) |  |  |  |
| Age (y), mean (SD) | 30.19 (9.18) | 31.27 (9.49) | -1.20 | .23 | 1078 |
| Gender identity, n (%) |  |  | 2.04 | .15 | 1 |
| Male | 113 (11.3) | 889 (88.7) |  |  |  |
| Transgender woman | 13 (16.7) | 65 (83.3) |  |  |  |
| Region, n (%) |  |  | 6.96 | .33 | 6 |
| Northeast | 11 (15.1) | 62 (84.9) |  |  |  |
| North | 29 (11.2) | 229 (88.8) |  |  |  |
| Central | 17 (12.6) | 118 (87.4) |  |  |  |
| East | 23 (8.9) | 235 (91.1) |  |  |  |
| South | 24 (13.6) | 153 (86.4) |  |  |  |
| Southwest | 19 (15.2) | 106 (84.8) |  |  |  |
| Northwest | 3 (5.6) | 51 (94.4) |  |  |  |
| Education level, n (%) |  |  | 0.70 | .40 | 1 |
| High school and below | 28 (13.3) | 182 (86.7) |  |  |  |
| High school above | 98 (11.3) | 772 (88.7) |  |  |  |
| Current employment status, n (%) |  |  | 2.57 | .28 | 2 |
| Full-time | 104 (12.4) | 736 (87.6) |  |  |  |
| Student | 13 (8.0) | 150 (92.0) |  |  |  |
| Unemployed or retired | 9 (11.7) | 68 (88.3) |  |  |  |
| Personal monthly income (yuan, 1 yuan=US $0.14) |  |  | 1.53 | .68 | 3 |
| <3000 | 26 (11.2) | 206 (88.8) |  |  |  |
| 3000-6999 | 49 (13.3) | 319 (86.7) |  |  |  |
| 7000-9999 | 20 (10.5) | 171 (89.5) |  |  |  |
| ≥10000 | 31 (10.7) | 258 (89.3) |  |  |  |
| Cohabitation status, n (%) |  |  | 2.49 | .65 | 4 |
| Alone | 51 (11.1) | 409 (88.9) |  |  |  |
| Partner | 17 (14.4) | 101 (85.6) |  |  |  |
| Family | 40 (12.6) | 278 (87.4) |  |  |  |
| Friends or roommates | 16 (9.3) | 156 (90.7) |  |  |  |
| Others | 2 (16.7) | 10 (83.3) |  |  |  |
| Sexual orientation, n (%) |  |  | 0.92 | .63 | 2 |
| Homosexuality | 92 (11.2) | 733 (88.8) |  |  |  |
| Bisexuality | 25 (13.2) | 165 (86.8) |  |  |  |
| Heterosexual and others | 9 (13.8) | 56 (86.2) |  |  |  |
| Sex role, n (%) |  |  | 4.93 | .85 | 2 |
| Receptive | 40 (12.5) | 281 (87.5) |  |  |  |
| Versatile | 35 (8.9) | 358 (91.1) |  |  |  |
| Insertive | 51 (13.9) | 315 (86.1) |  |  |  |
| Number of sexual partners, n (%) |  |  | 3.84 | .05 | 1 |
| 1 | 51 (14.4) | 303 (85.6) |  |  |  |
| ≥2 | 75 (10.3) | 651 (89.7) |  |  |  |
| Condom usage frequency during anal sex, n (%) |  |  | 0.77 | .86 | 3 |
| Every time | 65 (12.2) | 466 (87.8) |  |  |  |
| Often use | 28 (11.1) | 224 (88.9) |  |  |  |
| Occasionally use | 25 (11.9) | 185 (88.1) |  |  |  |
| Never use | 8 (9.2) | 79 (90.8) |  |  |  |
| Performed oral sex in the past 6 months, n (%) |  |  | 2.36 | .13 | 1 |
| No | 36 (14.4) | 214 (85.6) |  |  |  |
| Yes | 90 (10.8) | 740 (89.2) |  |  |  |
| Received oral sex in the past 6 months, n (%) |  |  | 0.21 | .64 | 1 |
| No | 47 (11.1) | 376 (88.9) |  |  |  |
| Yes | 79 (12.0) | 578 (88.0) |  |  |  |
| Condom usage frequency oral sex, n (%) |  |  | 5.05 | .16 | 4 |
| Every time | 7 (21.2) | 26 (78.8) |  |  |  |
| Often use | 2 (16.7) | 10 (83.3) |  |  |  |
| Occasionally use | 4 (6.2) | 61 (93.8) |  |  |  |
| Never use | 110 (11.9) | 816 (88.1) |  |  |  |
| No oral sex before | 3 (6.8) | 41 (93.2) |  |  |  |
| Had sex with regular partners in the past 6 months, n (%) |  |  | 0.05 | .82 | 1 |
| No | 53 (11.4) | 411 (88.6) |  |  |  |
| Yes | 73 (11.9) | 543 (88.1) |  |  |  |
| Had sex with temporary partners in the past 6 months, n (%) |  |  | 1.21 | .27 | 1 |
| No | 60 (12.9) | 405 (87.1) |  |  |  |
| Yes | 66 (10.7) | 549 (89.3) |  |  |  |
| Had commercial sex in the past 6 months, n (%) |  |  | 0.22 | .64 | 1 |
| No | 117 (11.5) | 896 (88.5) |  |  |  |
| Yes | 9 (13.4) | 58 (86.6) |  |  |  |
| Had group sex in the past 6 months, n (%) |  |  | 2.57 | .11 | 1 |
| No | 111 (12.4) | 786 (87.6) |  |  |  |
| Yes | 15 (8.2) | 168 (91.8) |  |  |  |
| Had sex with female in the past 6 months, n (%) |  |  | 0.46 | .49 | 1 |
| No | 110 (11.4) | 852 (88.6) |  |  |  |
| Yes | 16 (13.6) | 102 (86.4) |  |  |  |
| Had a circumcision, n (%) |  |  | 1.71 | .19 | 1 |
| No | 106 (12.3) | 755 (87.7) |  |  |  |
| Yes | 20 (9.1) | 199 (90.9) |  |  |  |
| Self-reported HIV status, n (%) |  |  | 4.72 | .09 | 2 |
| Negative | 91 (11.5) | 699 (88.5) |  |  |  |
| Positive | 7 (6.7) | 98 (93.3) |  |  |  |
| Unknown | 28 (15.1) | 157 (84.9) |  |  |  |
| History of STI, n (%) |  |  | 5.78 | .016 | 1 |
| No | 94 (13.4) | 608 (86.6) |  |  |  |
| Yes | 32 (8.5) | 346 (91.5) |  |  |  |
| Information (score), mean (SD) |  |  |  |  |  |
| Knowledge of HPV | 11.84 (6.19) | 13.66 (5.26) | -3.15 | .002 | 1078 |
| Motivation (score), mean (SD) |  |  |  |  |  |
| Perceived susceptibility | 2.89 (1.09) | 3.27 (1.00) | -3.94 | <.001 | 1078 |
| Perceived severity | 3.66 (1.06) | 3.81 (0.98) | -1.59 | .11 | 1078 |
| Perceived benefits | 4.05 (0.96) | 4.27 (0.80) | -2.79 | .005 | 1078 |
| Perceived barriers | 3.55 (0.98) | 3.30 (0.93) | 2.78 | .006 | 1078 |
| Subjective norms | 3.20 (0.85) | 3.47 (0.77) | -3.63 | <.001 | 1078 |
| Behavioral skills (score), mean (SD) |  |  |  |  |  |
| Decision-making | 3.78 (1.02) | 4.16 (0.86) | -3.98 | <.001 | 1078 |
| Self-efficacy | 3.61 (0.94) | 4.03 (0.80) | -4.78 | <.001 | 1078 |
| Objective skills | 3.60 (0.98) | 3.86 (0.87) | -2.79 | .006 | 1078 |

| Supplementary Table 5 Univariate analysis of factors influencing willingness for self-collected HPV testing among MSM in China (N=1080) | | | | |  |
| --- | --- | --- | --- | --- | --- |
| Variables | willingness for self-collected HPV testing | | Statistics | | |
|  | Unwillingness | willingness | *χ^2^ ort* | *P value* | *df* |
|  | (n=177) | (n=903) |  |  |  |
| Age (y), mean (SD) | 32.61 (9.80) | 30.85 (9.36) | 2.26 | .02 | 1078 |
| Gender identity, n (%) |  |  | 0.01 | .95 | 1 |
| Male | 164 (16.4) | 838 (83.6) |  |  |  |
| Transgender woman | 13 (16.7) | 65 (83.3) |  |  |  |
| Region, n (%) |  |  | 2.05 | .92 | 6 |
| Northeast | 13 (17.8) | 60 (82.2) |  |  |  |
| North | 45 (17.4) | 213 (82.6) |  |  |  |
| Central | 21 (15.6) | 114 (84.4) |  |  |  |
| East | 36 (14.0) | 222 (86.0) |  |  |  |
| South | 29 (16.4) | 148 (83.6) |  |  |  |
| Southwest | 23 (18.4) | 102 (81.6) |  |  |  |
| Northwest | 10 (18.5) | 44 (81.5) |  |  |  |
| Education level, n (%) |  |  | 5.79 | .01 | 1 |
| High school and below | 46 (21.9) | 164 (78.1) |  |  |  |
| High school above | 131 (15.1) | 739 (84.9) |  |  |  |
| Current employment status, n (%) |  |  | 12.57 | .002 | 2 |
| Full-time | 138 (16.4) | 702 (83.6) |  |  |  |
| Student | 17 (10.4) | 146 (89.6) |  |  |  |
| Unemployed or retired | 22 (28.6) | 55 (71.4) |  |  |  |
| Personal monthly income (yuan, 1 yuan=US $0.14) |  |  | 0.82 | .85 | 3 |
| <3000 | 38 (16.4) | 194 (83.6) |  |  |  |
| 3000-6999 | 62 (16.8) | 306 (83.2) |  |  |  |
| 7000-9999 | 34 (17.8) | 157 (82.2) |  |  |  |
| ≥10000 | 43 (14.9) | 246 (85.1) |  |  |  |
| Cohabitation status, n (%) |  |  | 9.51 | .05 | 4 |
| Alone | 72 (15.7) | 388 (84.3) |  |  |  |
| Partner | 24 (20.3) | 94 (79.7) |  |  |  |
| Family | 55 (17.3) | 263 (82.7) |  |  |  |
| Friends or Roommates | 21 (12.2) | 151 (87.8) |  |  |  |
| Others | 5 (41.7) | 7 (58.3) |  |  |  |
| Sexual orientation, n (%) |  |  | 0.22 | .90 | 2 |
| Homosexuality | 134 (16.2) | 691 (83.8) |  |  |  |
| Bisexuality | 31 (16.3) | 159 (83.7) |  |  |  |
| Heterosexual and others | 12 (18.5) | 53 (81.5) |  |  |  |
| Sex role, n (%) |  |  | 0.80 | .67 | 2 |
| Receptive | 57 (17.8) | 264 (82.2) |  |  |  |
| Versatile | 60 (15.3) | 333 (84.7) |  |  |  |
| Insertive | 60 (16.4) | 306 (83.6) |  |  |  |
| Number of sexual partners, n (%) |  |  | 0.49 | .49 | 1 |
| 1 | 62 (17.5) | 292 (82.5) |  |  |  |
| ≥2 | 115 (15.8) | 611 (84.2) |  |  |  |
| Condom usage frequency during anal sex, n (%) |  |  | 0.44 | .93 | 3 |
| Every time | 85 (16.0) | 446 (84.0) |  |  |  |
| Often use | 42 (16.7) | 210 (83.3) |  |  |  |
| Occasionally use | 37 (17.6) | 173 (82.4) |  |  |  |
| Never use | 13 (14.9) | 74 (85.1) |  |  |  |
| Performed oral sex in the past 6 months, n (%) |  |  | 0.96 | .33 | 1 |
| No | 46 (18.4) | 204 (81.6) |  |  |  |
| Yes | 131 (15.8) | 699 (84.2) |  |  |  |
| Received oral sex in the past 6 months, n (%) |  |  | 0.01 | .91 | 1 |
| No | 70 (16.5) | 353 (83.5) |  |  |  |
| Yes | 107 (16.3) | 550 (83.7) |  |  |  |
| Condom usage frequency oral sex, n (%) |  |  | 5.34 | .25 | 4 |
| Every time | 7 (21.2) | 26 (78.8) |  |  |  |
| Often use | 1 (8.3) | 11 (91.7) |  |  |  |
| Occasionally use | 16 (24.6) | 49 (75.4) |  |  |  |
| Never use | 144 (15.6) | 782 (84.4) |  |  |  |
| No oral sex before | 9 (20.5) | 35 (79.5) |  |  |  |
| Had sex with regular partners in the past 6 months, n (%) |  |  | 0.03 | .87 | 1 |
| No | 77 (16.6) | 387 (83.4) |  |  |  |
| Yes | 100 (16.2) | 516 (83.8) |  |  |  |
| Had sex with temporary partners in the past 6 months, n (%) |  |  | 0.01 | .97 | 1 |
| No | 76 (16.3) | 389 (83.7) |  |  |  |
| Yes | 101 (16.4) | 514 (83.6) |  |  |  |
| Had commercial sex in the past 6 months, n (%) |  |  | 0.01 | .99 | 1 |
| No | 166 (16.4) | 847 (83.6) |  |  |  |
| Yes | 11 (16.4) | 56 (83.6) |  |  |  |
| Had group sex in the past 6 months, n (%) |  |  | 0.01 | .99 | 1 |
| No | 147 (16.4) | 750 (83.6) |  |  |  |
| Yes | 30 (16.4) | 153 (83.6) |  |  |  |
| Had sex with female in the past 6 months, n (%) |  |  | 0.93 | .34 | 1 |
| No | 154 (16.0) | 808 (84.0) |  |  |  |
| Yes | 23 (19.5) | 95 (80.5) |  |  |  |
| Had a circumcision, n (%) |  |  | 1.00 | .32 | 1 |
| No | 146 (17.0) | 715 (83.0) |  |  |  |
| Yes | 31 (14.2) | 188 (85.8) |  |  |  |
| Self-reported HIV status, n (%) |  |  | 3.23 | .20 | 2 |
| Negative | 125 (15.8) | 665 (84.2) |  |  |  |
| Positive | 14 (13.3) | 91 (86.7) |  |  |  |
| Unknown | 38 (20.5) | 147 (79.5) |  |  |  |
| History of STI, n (%) |  |  | 0.03 | .86 | 1 |
| No | 114 (16.2) | 588 (83.8) |  |  |  |
| Yes | 63 (16.7) | 315 (83.3) |  |  |  |
| Information (score), mean (SD) |  |  |  |  |  |
| Knowledge of HPV | 12.64 (5.95) | 13.6 (5.28) | -1.99 | .04 | 1078 |
| Motivation (score), mean (SD) |  |  |  |  |  |
| Perceived susceptibility | 3.11 (1.06) | 3.25 (1.00) | -1.69 | .09 | 1078 |
| Perceived severity | 3.63 (1.08) | 3.82 (0.97) | -2.24 | .03 | 1078 |
| Perceived benefits | 4.06 (0.94) | 4.28 (0.79) | -3.24 | .001 | 1078 |
| Perceived barriers | 3.20 (0.96) | 3.36 (0.93) | -2.03 | .04 | 1078 |
| Subjective norms | 3.35 (0.83) | 3.46 (0.77) | -1.64 | .10 | 1078 |
| Behavioral skills (score), mean (SD) |  |  |  |  |  |
| Decision-making | 4.00 (0.98) | 4.13 (0.86) | -1.83 | .07 | 1078 |
| Self-efficacy | 3.90 (0.90) | 4.00 (0.81) | -1.33 | .19 | 1078 |
| Objective skills | 3.66 (0.92) | 3.86 (0.88) | -2.75 | .006 | 1078 |

| Supplementary Table 6 Univariate analysis of factors influencing willingness for regular HPV testing among MSM in China (N=1080) | | | | |  |
| --- | --- | --- | --- | --- | --- |
| Variables | willingness for regular HPV testing | | Statistics | | |
|  | Unwillingness | willingness | *χ^2^ or t* | *P value* | *df* |
|  | (n=230) | (n=850) |  |  |  |
| Age (y), mean (SD) | 30.71 (9.27) | 31.26 (9.50) | -0.78 | .43 | 1078 |
| Gender identity, n (%) |  |  | 3.37 | .07 | 1 |
| Male | 207 (20.7) | 795 (79.3) |  |  |  |
| Transgender woman | 23 (29.5) | 55 (70.5) |  |  |  |
| Region, n (%) |  |  | 5.22 | .52 | 6 |
| Northeast | 12 (16.4) | 61 (83.6) |  |  |  |
| North | 46 (17.8) | 212 (82.2) |  |  |  |
| Central | 31 (23) | 104 (77) |  |  |  |
| East | 64 (24.8) | 194 (75.2) |  |  |  |
| South | 40 (22.6) | 137 (77.4) |  |  |  |
| Southwest | 26 (20.8) | 99 (79.2) |  |  |  |
| Northwest | 11 (20.4) | 43 (79.6) |  |  |  |
| Education level, n (%) |  |  | 0.26 | .61 | 1 |
| High school and below | 42 (20.0) | 168 (80.0) |  |  |  |
| High school above | 188 (21.6) | 682 (78.4) |  |  |  |
| Current employment status, n (%) |  |  | 1.81 | .40 | 2 |
| Full-time | 174 (20.7) | 666 (79.3) |  |  |  |
| Student | 35 (21.5) | 128 (78.5) |  |  |  |
| Unemployed or retired | 21 (27.3) | 56 (72.7) |  |  |  |
| Personal monthly income (yuan, 1 yuan=US $0.14) |  |  | 2.30 | .51 | 3 |
| <3000 | 56 (24.1) | 176 (75.9) |  |  |  |
| 3000-6999 | 80 (21.7) | 288 (78.3) |  |  |  |
| 7000-9999 | 35 (18.3) | 156 (81.7) |  |  |  |
| ≥10000 | 59 (20.4) | 230 (79.6) |  |  |  |
| Cohabitation status, n (%) |  |  | 9.25 | .06 | 4 |
| Alone | 79 (17.2) | 381 (82.8) |  |  |  |
| Partner | 31 (26.3) | 87 (73.7) |  |  |  |
| Family | 77 (24.2) | 241 (75.8) |  |  |  |
| Friends or Roommates | 39 (22.7) | 133 (77.3) |  |  |  |
| Others | 4 (33.3) | 8 (66.7) |  |  |  |
| Sexual orientation, n (%) |  |  | 2.38 | .30 | 2 |
| Homosexuality | 181 (21.9) | 644 (78.1) |  |  |  |
| Bisexuality | 33 (17.4) | 157 (82.6) |  |  |  |
| Heterosexual and others | 16 (24.6) | 49 (75.4) |  |  |  |
| Sex role, n (%) |  |  | 0.96 | .62 | 2 |
| Receptive | 66 (20.6) | 255 (79.4) |  |  |  |
| Versatile | 90 (22.9) | 303 (77.1) |  |  |  |
| Insertive | 74 (20.2) | 292 (79.8) |  |  |  |
| Number of sexual partners, n (%) |  |  | 0.14 | .71 | 1 |
| 1 | 73 (20.6) | 281 (79.4) |  |  |  |
| ≥2 | 157 (21.6) | 569 (78.4) |  |  |  |
| Condom usage frequency during anal sex, n (%) |  |  | 4.78 | .19 | 3 |
| Every time | 102 (19.2) | 429 (80.8) |  |  |  |
| Often use | 54 (21.4) | 198 (78.6) |  |  |  |
| Occasionally use | 49 (23.3) | 161 (76.7) |  |  |  |
| Never use | 25 (28.7) | 62 (71.3) |  |  |  |
| Performed oral sex in the past 6 months, n (%) |  |  | 0.05 | .83 | 1 |
| No | 52 (20.8) | 198 (79.2) |  |  |  |
| Yes | 178 (21.4) | 652 (78.6) |  |  |  |
| Received oral sex in the past 6 months, n (%) |  |  | ＜0.01 | .99 | 1 |
| No | 90 (21.3) | 333 (78.7) |  |  |  |
| Yes | 140 (21.3) | 517 (78.7) |  |  |  |
| Condom usage frequency oral sex, n (%) |  |  | 0.70 | .95 | 4 |
| Every time | 8 (24.2) | 25 (75.8) |  |  |  |
| Often use | 2 (16.7) | 10 (83.3) |  |  |  |
| Occasionally use | 15 (23.1) | 50 (76.9) |  |  |  |
| Never use | 197 (21.3) | 729 (78.7) |  |  |  |
| No oral sex before | 8 (18.2) | 36 (81.8) |  |  |  |
| Had sex with regular partners in the past 6 months, n (%) |  |  | 4.54 | .03 | 1 |
| No | 113 (24.4) | 351 (75.6) |  |  |  |
| Yes | 117 (19.0) | 499 (81.0) |  |  |  |
| Had sex with temporary partners in the past 6 months, n (%) |  |  | 3.26 | .07 | 1 |
| No | 87 (18.7) | 378 (81.3) |  |  |  |
| Yes | 143 (23.3) | 472 (76.7) |  |  |  |
| Had commercial sex in the past 6 months, n (%) |  |  | 0.49 | .49 | 1 |
| No | 218 (21.5) | 795 (78.5) |  |  |  |
| Yes | 12 (17.9) | 55 (82.1) |  |  |  |
| Had group sex in the past 6 months, n (%) |  |  | 0.15 | .70 | 1 |
| No | 193 (21.5) | 704 (78.5) |  |  |  |
| Yes | 37 (20.2) | 146 (79.8) |  |  |  |
| Had sex with female in the past 6 months, n (%) |  |  | 0.72 | .79 | 1 |
| No | 206 (21.4) | 756 (78.6) |  |  |  |
| Yes | 24 (20.3) | 94 (79.7) |  |  |  |
| Had a circumcision, n (%) |  |  | 1.51 | .22 | 1 |
| No | 190 (22.1) | 671 (77.9) |  |  |  |
| Yes | 40 (18.3) | 179 (81.7) |  |  |  |
| Self-reported HIV status, n (%) |  |  | 2.65 | .27 | 2 |
| Negative | 168 (21.3) | 622 (78.7) |  |  |  |
| Positive | 17 (16.2) | 88 (83.8) |  |  |  |
| Unknown | 45 (24.3) | 140 (75.7) |  |  |  |
| History of STI, n (%) |  |  | 1.03 | .31 | 1 |
| No | 156 (22.2) | 546 (77.8) |  |  |  |
| Yes | 74 (19.6) | 304 (80.4) |  |  |  |
| Information (score), mean (SD) |  |  |  |  |  |
| Knowledge of HPV | 12.71 (5.61) | 13.64 (5.32) | -2.32 | .02 | 1078 |
| Motivation (score), mean (SD) |  |  |  |  |  |
| Perceived susceptibility | 3.08 (1.06) | 3.27 (1.00) | -2.46 | .01 | 1078 |
| Perceived severity | 3.78 (1.05) | 3.79 (0.97) | -0.25 | .80 | 1078 |
| Perceived benefits | 4.28 (0.80) | 4.24 (0.83) | 0.67 | .50 | 1078 |
| Perceived barriers | 3.39 (0.94) | 3.31 (0.94) | 1.17 | .24 | 1078 |
| Subjective norms | 3.36 (0.81) | 3.46 (0.77) | -1.77 | .08 | 1078 |
| Behavioral skills (score), mean (SD) |  |  |  |  |  |
| Decision-making | 4.05 (0.89) | 4.13 (0.88) | -1.14 | .25 | 1078 |
| Self-efficacy | 3.95 (0.80) | 3.99 (0.83) | -0.70 | .49 | 1078 |
| Objective skills | 3.80 (0.86) | 3.83 (0.90) | -0.57 | .57 | 1078 |
